# Long-lived tissue resident HIV-1 specific memory CD8^+^ T cells are generated by skin immunization with live virus vectored microneedle arrays

**DOI:** 10.1016/j.jconrel.2017.10.026

**Published:** 2017-12-28

**Authors:** Marija Zaric, Pablo Daniel Becker, Catherine Hervouet, Petya Kalcheva, Barbara Ibarzo Yus, Clement Cocita, Lauren Alexandra O'Neill, Sung-Yun Kwon, Linda Sylvia Klavinskis

**Affiliations:** a Peter Gorer Department of Immunobiology, Faculty of Life Sciences and Medicine, King's College London, London SE1 9RT, United Kingdom; b TheraJect Inc., Fremont, CA 94538, United States

**Keywords:** Microneedles, Viral vector, Tissue resident CD8, Memory, Mucosal tissue, HIV

## Abstract

The generation of tissue resident memory (T_RM_) cells at the body surfaces to provide a front line defence against invading pathogens represents an important goal in vaccine development for a wide variety of pathogens. It has been widely assumed that local vaccine delivery to the mucosae is necessary to achieve that aim. Here we characterise a novel micro-needle array (MA) delivery system fabricated to deliver a live recombinant human adenovirus type 5 vaccine vector (AdHu5) encoding HIV-1 gag. We demonstrate rapid dissolution kinetics of the microneedles in skin. Moreover, a consequence of MA vaccine cargo release was the generation of long-lived antigen-specific CD8^+^ T cells that accumulate in mucosal tissues, including the female genital and respiratory tract. The memory CD8^+^ T cell population maintained in the peripheral mucosal tissues was attributable to a MA delivered AdHu5 vaccine instructing CD8^+^ T cell expression of CXCR3^+^, CD103^+,^ CD49a^+^, CD69^+^, CD127^+^ homing, retention and survival markers. Furthermore, memory CD8^+^ T cells generated by MA immunization significantly expanded upon locally administered antigenic challenge and showed a predominant poly-functional profile producing high levels of IFNγ and Granzyme B. These data demonstrate that skin vaccine delivery using microneedle technology induces mobilization of long lived, poly-functional CD8^+^ T cells to peripheral tissues, phenotypically displaying hallmarks of residency and yields new insights into how to design and deliver effective vaccine candidates with properties to exert local immunosurveillance at the mucosal surfaces.

## Graphical abstract

**Figure:**
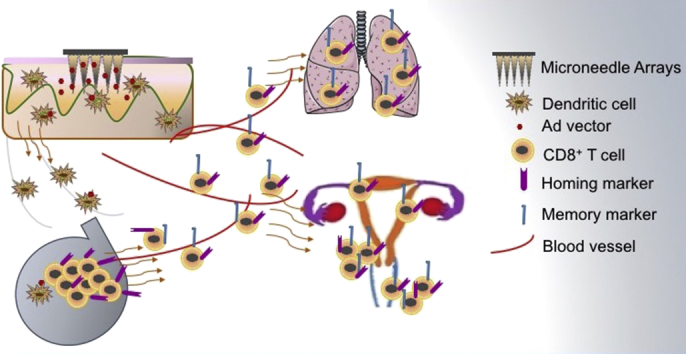

## 1 Introduction

Human immunodeficiency virus (HIV) remains a global health threat, and no HIV vaccine developed to date has achieved prolonged or significant protection in humans. Therefore, the development of a safe and effective HIV vaccine for prophylactic and therapeutic use represents not only an unprecedented scientific challenge but also a fundamental necessity to redress the economic and social impact of infection [1]. While intense efforts have been directed to develop vaccines that provide protective antibody responses against HIV [1], it is equally acknowledged that antigen-specific memory CD8^+^ T cells contribute a critical, complementary role in the control of HIV-1 infection [1], [2], [3]. It is currently understood that memory CD8^+^ T cells provide a multi-layered protective immune response, by residing in different anatomic niches, as lymphoid tissue-based ‘central’ memory CD8^+^ T-cells (T_CM_), as circulating effector memory CD8^+^ T-cells (T_EM_) that patrol non-lymphoid tissues and as non-lymphoid tissue resident memory T cells (T_RM_) [4], [5], [6]. Each subset express distinct phenotypic markers and contribute distinct roles in immuno-surveillance of the host [4], [5], [6]. As a consequence there is an unmet need to develop vaccine strategies that generate memory CD8^+^ T cells at each of these anatomic niches as an integrated immune surveillance network against mucosally acquired pathogens.

Under physiological conditions, T_RM_ cells by virtue of their residence in epithelial barrier tissues at the interface between the host and the environment, such as the skin, gastrointestinal, respiratory and reproductive tracts can respond rapidly to pathogen challenge at these sites, independently of T cell recruitment from the blood [7], [8]. They thus mediate the rapid protective immunity that is the hallmark of adaptive immune memory [7]. Therefore, vaccination strategies that can in addition programme antigen experienced T cells to provide long term memory at the mucosal surfaces and respond rapidly to antigen re-encounter, would be of immense benefit in the development of effective vaccines against mucosally acquired pathogens, including HIV.

The skin is an attractive target for vaccine delivery for ease of administration and as a consequence of the high density of antigen presenting cells localized in the epidermis and dermis that are accessible for acquisition of vaccine antigens [9], [10], [11], [12]. Current vaccination strategies involve the use of intra-dermal needle injections as a system for vaccine delivery to this rich network of cutaneous antigen-presenting cells. However, there are numerous drawbacks with this mode of vaccination including the need for trained staff, pain/bleeding associated with the injection itself, the requirement for safe needle disposal procedures and the risk of accidental injury or inappropriate needle reuse. Moreover, in resource-limited settings, an additional level of consideration is the need to maintain a continuous ‘cold chain’ for vaccine storage and transport to prevent any loss in potency. These downsides have led to the development of new designs for vaccine delivery such as microneedle arrays (MA), that are not only safe, cost-effective and thermostable, but most importantly proven to elicit systemic and mucosal immunity and improve vaccine efficacy [13], [14].

MAs fabricated from a dissolvable polymer matrix to contain viral vector based vaccines, including adenovirus vectors, are at the forefront of recent technological advances that successfully achieve live vector thermostability with a delivery platform that exploits the rich antigen presenting cell network in the skin to induce potent cellular immune responses [13], [15], [16]. We have previously reported the fabrication of MAs from sodium carboxymethylcellulose (Na-CMC), an FDA approved injectable bio-material, that is inert, non-toxic, free of mutagenic or carcinogenic properties and has been used as a GMP reagent in the clinic over the last 50 years [17]. By incorporating a disaccharide in the Na-CMC MA matrix, we reported the thermal stabilisation of adenovirus vectors at ambient temperature, with no significant loss in immunogenicity when administered to mice as determined by induction of high frequency of antigen-specific CD8 T cells [13]. In vivo confocal microscopy of MAs delivered to CD11c eGFP reporter mice demonstrated dissolution of the MA cargo following penetration of mouse skin and uptake by CD11c^+^ antigen presenting cells in the dermis [13]. Moreover, MA vaccination with live, recombinant adenovirus (Ad) virus vectors prime high-frequency effector CD8^+^ T cells that express multi-functional cytokines, detected in systemic and mucosal associated lymphoid tissues [13], [16]. However, the capacity of MA vaccination to programme antigen experienced T cells to provide long term memory, especially at the mucosal surfaces and respond rapidly to antigen re-encounter which is the hallmark of a vaccine, remains largely unexplored.

In this study, we demonstrate that a simple, thermostable MA, fabricated to contain a recombinant human adenovirus type 5 vector (rAd5Hu) encoding HIV- 1 gag, rapidly dissolves in skin following application and generated robust antigen-specific CD8^+^ T cells in the blood and non–lymphoid mucosal tissues, including the genital and respiratory tracts. Moreover, vaccine elicited CD8^+^ memory T cells were retained in peripheral tissues for at least one-year post-vaccination and critically, retained the functional capacity to respond to secondary antigen challenge by production of antiviral effector molecules. As the induction of robust T_RM_ responses at the barrier surfaces represents an imperative goal for an effective HIV vaccine, this study demonstrates the promising potential of an accessible MA skin immunization platform to elicit memory T_RM_ as part of a multi-layered protective immune response.

## 2 Material and methods

### 2.1 Recombinant adenovirus vaccine

A recombinant E1, E3 deleted human adenovirus type 5 (AdHu5) vaccine vector encoding HIV-1 CN54gag (AdHu5-CN54gag) was used which has been described previously [13]. High titre virus stocks were prepared on AD293 cells (Cell Biolabs, Inc., San Diego, USA) and purified by the Native Antigen Company (Oxford, UK). Viruses were tested for endotoxin using the Kinetic-QCL assay (Lonza, Slough, UK), according to the manufacturer's instructions.

### 2.2 Adenovirus titres

Virus particle titre (vp) based on viral genome equivalence was determined using the Quant-it PicoGreen dsDNA assay kit (Invitrogen). Virus samples were denatured for 30 min at 55 °C in 0.05% sodium dodecyl sulphate (SDS, Sigma) containing Tris-EDTA (TE, Sigma). Virus DNA binding to fluorescent PicoGreen was measured at 485 nm excitation wavelength (λ ex) and 535 nm emission wavelength (λ em) using the PicoGreen FluroScanner™ with Ascent 2.5 software. Calculation of virus titre in particles per ml was computed from the extrapolated concentration multiplied by the dilution factor and particle number factor, which was determined as 2.5 × 10^10^ for Ad5, as described previously [18].

### 2.3 Fabrication of microneedle arrays (MA)

Dissolvable microneedles (1500 μm in length, 670 μm in base diameter and 44 per array) were fabricated by a centrifugation casting method using an inverted cone shaped silicone template as described previously [13]. In brief, the needle tips were prepared from sodium carboxymethylcellulose (Na-CMC), a biocompatible, highly water-soluble polymer to provide mechanical strength and sucrose, as a protein stabilizer [13]. AdHu5-CN54gag was formulated in the matrix of the needle tips. Additional mechanical strength was added by layering a second matrix (12% Na-CMC, 4.8% lactose) to create the needle shaft and finally a pre-made membrane (8% Na-CMC, 0.8% lactose) applied to the needle base.

### 2.4 immunization of mice

C57BL/6 (B6), 6–8 weeks old, female mice of H-2b haplotype were purchased from Envigo. MAs were applied manually with gentle pressure to the dorsal skin of mice, shaved of hair and depilated with hair removal cream (Veet™) as described previously [13]. All animal husbandry and experimentation was approved under a project license granted by the United Kingdom Home Office.

### 2.5 Isolation of cells from blood and tissues

In some experiments, blood was collected from the tail vein into EDTA-coated Eppendorf tubes and further treated with red blood cell lysis buffer (Sigma–Aldrich) prior to Ab staining. Lungs and female genital tracts (GTs) were harvested from perfused female mice and broncho-alveolar lavage (BAL) fluid was also collected where indicated as described previously [19].

Single cell suspensions from the spleen were prepared in complete RPMI medium (RPMI 1640) supplemented with 100 U/ml penicillin, 100 μg/ml streptomycin, 2 mM l-glutamine (Invitrogen), 50 μM 2-mercaptoethanol (Invitrogen) and 10% heat inactivated FBS (Biosera). Erythrocytes were depleted by treatment with red blood cell lysis buffer (Sigma–Aldrich). Single cell suspensions from the mucosal tissues were obtained by enzymatic digestion for 30 min at 37 °C in complete RPMI 1640 containing 1 mg/ml Collagenase D (RocheTM) and 0.02 mg/ml DNase I from bovine pancreas (RocheTM).

### 2.6 Antibodies and reagents for flow cytometry

The following antibodies were purchased from BD Bio-sciences: purified anti-Fc RII/III mAb (CD16/32, 2.4G2), anti-CD8 (53–6.7), anti-CD4 (RM4-5), anti-CD3 (145-2C11), anti-CD62L (MEL-14), anti-CD127 (SB/199), anti-KLRG-1 (2F1), anti-CD103 (2E7), anti-CD49a (HMα1), anti-CD69 (H1.2F3), anti-CXCR3 (CXCR3-173) and anti-CD45.2 (104). In addition, anti-IFNγ (XMG1.2) and anti-Granzyme B (NGZB) were obtained from eBioscience, and anti-MHCII (M5/114.15.2) from Biolegend. Flurophore-conjugated tetramer to the immunodominant H-2Db restricted HIV-1 CN54 gag _308–318_ epitope (Db/CN54gag) for identification of HIV-1 CN54gag specific CD8 T cells as described previously [13] was kindly synthesized by the NIH Tetramer core facility (Emory University, USA).

### 2.7 Cell staining for flow cytometry

Cells were stained with the Db/CN54gag tetramer for 15 min at room temperature, followed by addition of cell surface antibodies (20 min) and then re-washed.

For intracellular cytokine staining, cells were washed, counted, resuspended at 1 × 10^6^/ml and restimulated in RPMI 10% medium at 37 °C for 4 h with 50 ng/ml phorbol myristate acetate (PMA, Sigma-Aldrich), 500 ng/ml Ionomycin (Sigma-Aldrich) in the presence of Brefeldin A (Golgi Plug, BD Biosciences). Following surface staining, cells were fixed and permeabilized by incubating in BD CytoFix-CytoPerm for 20 min in the dark at 4 °C. The cells were then washed twice with Perm/Fix wash buffer and re-suspended in 50 μl of the BD Perm/wash buffer (BD Pharmingen™) with fluorochrome conjugated monoclonal antibody and incubated for 30 min in the dark at 4 °C. Cells were then washed twice in Perm/wash buffer prior to flow cytometry analyses.

Samples were acquired using a BD FACSCanto II or BD SORP Fortessa™ flow cytometers and analyzed using FlowJo software version 9.7.5 (Tree Star).

### 2.8 Vaginal antigen challenge

150 days post MA immunization, mice were injected subcutaneously with 3 mg of Depo-Provera (Pfizer), a long-lasting progestin, in 100 μL of sterile PBS 5 days before intravaginal challenge. Groups of mice were challenged by depositing 20 μg of HIV-1 CN54 gag_308–318_ peptide (ProImmune) and 30μg of CpG ODN 1826 (InvivoGen) into vaginal wall and monitored for 5 days.

### 2.9 Statistical analysis

Results were analyzed with GraphPad PRISM™ version 6.0 (San Diego, Ca, USA). Comparisons between groups were performed using a one-way ANOVA in conjunction with Bonferroni post analyses test. Data are shown as mean ± SEM. Probability values are expressed as following: ***P < 0.001, **P < 0.01 and *P < 0.05.

## 3 Results

### 3.1 Microneedle Array (MA) mediated skin delivery

**Fig. 1:**
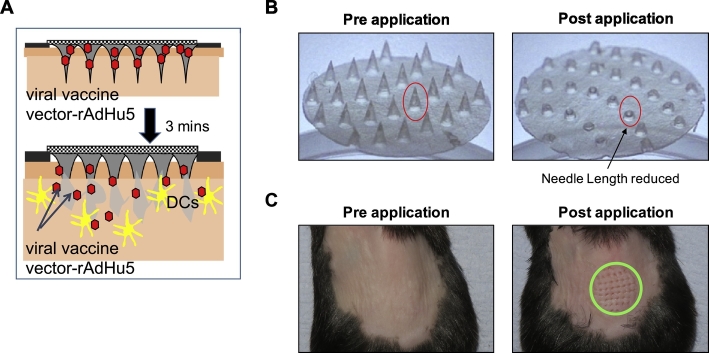
Microneedle Array (MA) mediated skin delivery. (A) Schematic representation of the mechanism of action of the dissolving MA. (B) MA fabricated with rAdHu5-CN54gag in a sucrose-Na-CMC matrix pre-(left) and post-(right) application. (C) En face view of B6 back skin (left) before and (right) 3 min after MA application.

To evaluate the potential of a dried rAdHu5-CN54gag vaccine in a needle-free delivery system, dissolvable microneedles (1500 μm in length, 44 per array) were fabricated within a silicone template. A Na-CMC-sucrose matrix blended with virus formed the needle tips (Fig. 1A). Mechanical strength was added by layering a second matrix [12% (wt/vol) Na-CMC, 4.8% (wt/vol) lactose] creating the needle shaft and applying a premade membrane [8% (wt/vol) Na-CMC, 0.8% lactose) to the needle base. Each MA patch in total contained an average of 8 × 10^8^ virus particles (vp) of rAdHu5 CN54gag determined by virus recovery. The MAs were mechanically strong and punctured the back skin of B6 mice when gentle pressure was applied to the MA base plate. At the same time, the MA composition facilitated rapid dissolution. Representative images of a MA patch prior to application and following removal from the skin (Fig. 1B) illustrate that a reduction in needle length by approximately two-thirds is achieved within 3 min of application (Fig. 1B). Coincident with removal of the MA patch, micron scale punctures within the skin corresponding to the array template were observed (Fig. 1C). These data indicate that the MA design has the capacity to delivery a vaccine cargo into the skin.

### 3.2 In vivo vaccine release profile from MAs encapsulating rAdHu5-CN54gag

**Fig. 2:**
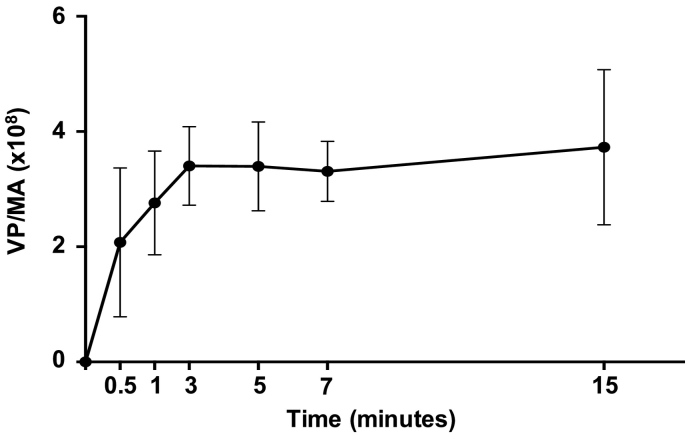
In vivo vaccine release profile from microneedle arrays encapsulating a rAdHu5 encoding HIV-1 CN54 gag_._ MAs containing a rAdHu5 vector encoding HIV-1 CN54 gag within a water-soluble matrix were applied to murine back skin over a time course of 0.5 to 15 min. After application, the remaining MA base-plates were dissolved in PBS to release residual encapsulated virus and rAdHu5-CN54gag titre quantitated by PicoGreen assay as virus particle number (vp). rAdHu5-CN54gag delivered to the skin per single MA was calculated as the difference in MA vp number before and after application. Data are pooled from two independent experiments, *n* = 6–10 per each time point.

We next sought to determine the kinetics of rAd5Hu vectored vaccine release in B6 mice following MA application to skin, because such information is germane to establishing the most effective and consistent delivery schedule necessary to evaluate the immunogenic capacity of this delivery platform in this model system. To that end, MAs fabricated to contain a rAd5Hu-CN54gag vaccine were applied to dorsal skin of B6 mice over a time course from 0.5 to 15 min. Vaccine release was quantified as the mean difference in Ad5 virus particle titre per MA before application to that remaining in the MA base plate after retrieval from the skin. The release profile was rapid; an initial release was determined within 0.5 min and increased over 3 min of MA application in the skin. Extended application beyond 3 min did not increase the amount of virus released from the encapsulating MA matrix (Fig. 2). Therefore, we established that a 3 min application was sufficient to optimally deliver virus from this MA formulation onto the dorsal skin of B6 mice.

### 3.3 Ag-specific CD8^+^ T cells are elicited by MA immunization and express homing/retention markers in the female genital tract

**Fig. 3:**
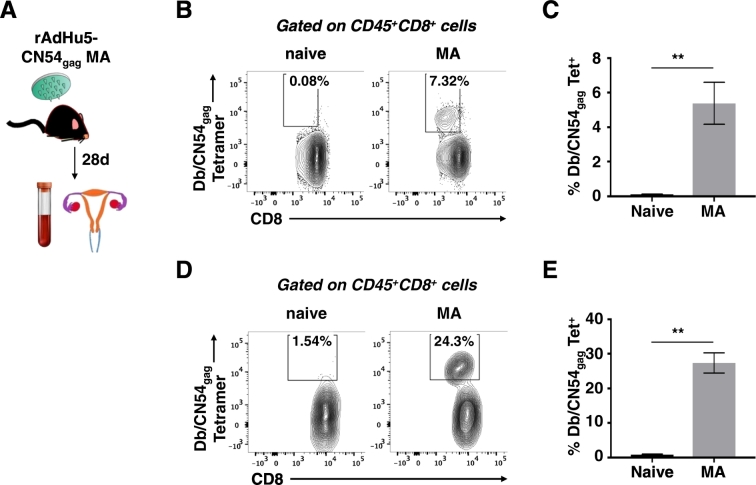
MA delivery of a rAdHu5 vector encoding HIV-1 CN54 gag induces systemic and mucosal CD8^+^ T cell memory responses. (A) Female C57BL/6 mice were immunized with rAdHu5-CN54 gag virus by dried MA application to dorsal skin or left naïve. Blood and genital tract tissues were collected 28 days post immunization for flow cytometric analysis. Frequency of Db/CN54gag tetramer expressing cells in blood (B) and genital tract (D) induced by MA immunization is shown from a gated CD45^+^ CD8^+^ population. Values (B and D) represent percentage of cells in each gate and are representative of four naïve and nine vaccinated mice from two independent experiments. (C and E); Bar graphs represent the frequency of Db/CN54gag Tetramer^+^ CD8^+^ T cells in blood (C) and genital tract (E) of rAdHu5-CN54 gag MA immunized mice.

Previously we established that MA immunization elicits primary effector T cell responses equipotent with conventional injectable routes of vaccine delivery [13]. As a next step, it was incumbent to examine the capacity of the dried rAdHu5-CN54gag MA platform to induce durable immune responses. Here we focused on CD8^+^ T cell immunity, acknowledged to be highly desirable in the context of HIV control [20]. Groups of B6 mice were immunized by rHuAd5-CN54gag MA application to the skin and antigen-specific CD8^+^ T-cell expansion tracked by tetramer to the immunodominant Db-restricted HIV-1 CN54 gag_308–318_ epitope (Db/CN54gag) in blood and genital tract tissues at day 28 post immunization (Fig. 3A). A substantial population of Db/CN54gag tetramer^+^ CD8^+^ T cells was detected in the systemic compartment (5–7%) (Fig. 3B and C) and importantly also in the GT of immunized female mice (20–30%) (Fig. 3D and E).

**Fig. 4:**
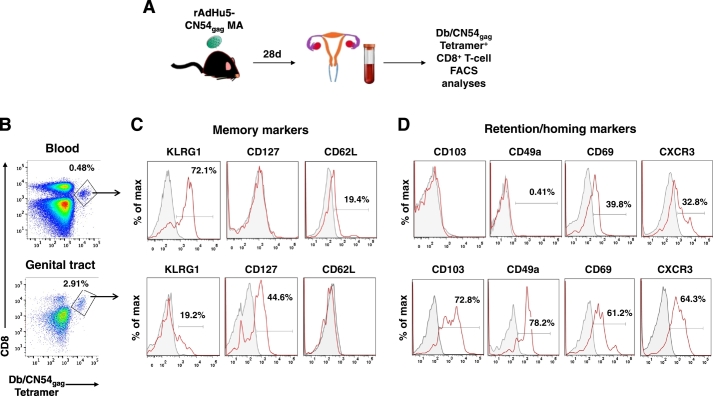
Memory CD8^+^ T cells elicited by MA vaccination express peripheral tissue homing and retention and markers in the female genital tract. (A) Db/CN54gag tetramer^+^ CD8^+^ T-cells were assessed in the blood and genital tracts of female B6 mice 28 days post immunization with rAdHu5-CN54gag MA. (B) Dot plots of Db/CN54gag-specific CD8^+^ T cells in blood (lower panel) and genital tracts (upper panel) 28 days after MA immunization with rAdHu5-CN54 gag. (C) Representative histograms indicating the frequency of CD8^+^ T cell differentiation/memory markers (KLRG1, CD127 and CD62L) expressed by Db/CN54gag tetramer^+^ CD8^+^ T cells (red lines) isolated from blood (lower panels) or genital tracts (upper panels) of rAdHu5-CN54 gag MA immunized mice. Grey histograms represent isotype control staining. (D) Representative histograms indicating the frequency of CD103, CD49a, CD69 or CXCR3 expressing Db/CN54gag tetramer^+^ CD8^+^ T cells (red lines) isolated from blood (lower panels) or genital tracts (upper panels) of rAdHu5-CN54gag MA immunized mice. Grey histograms represent staining with isotype controls. Data are representative of four naïve and nine vaccinated mice from two independent experiments.

As skin MA immunization induced Ag-specific CD8^+^ T cells localized in mucosal non-lymphoid tissue at day 28 after immunization, we analyzed tetramer-specific CD8^+^ T cells in the blood and infiltrating the GT for markers that instruct memory CD8 T cells to home, to be retained and survive in non-lymphoid tissue (Fig. 4A). Db/CN54gag tetramer^+^ CD8^+^ T cells infiltrating the GT (Fig. 4B) showed increased expression of an early memory/homeostatic survival marker CD127 [21], relative to that expressed by the circulating systemic pool of Db/CN54gag tetramer^+^ CD8^+^ T cells. In sharp contrast, expression of killer lectin receptor G1 (KLRG1) a marker of activated terminally differentiated cells [22] was considerably lower on Db/CN54gag tetramer^+^ CD8^+^ T cells infiltrating the GT (19.2%) when cross compared to Db/CN54gag tetramer^+^ CD8^+^ T cells isolated from the systemic circulation (72.1%; Fig. 4C). Likewise, Db/CN54gag tetramer^+^ CD8^+^ T cells infiltrating the GT did not express CD62L, a lymphoid tissue trafficking receptor (Fig. 4B and C). Consistent with this observation, GT infiltrating CD8^+^ T cells demonstrated a substantial increase in expression of markers associated with non-lymphoid tissue residency, CD49a, CD69 and CD103, representing 78.2%, 61.2% and 72.8% of the Db/CN54gag tetramer^+^ CD8^+^ population respectably as compared in the blood (0.41%, 39.8% and 0% of the Db/CN54gag tetramer^+^ CD8^+^ population respectably (Fig. 4D). Noticeably, the expression of CXCR3, a chemokine receptor that is highly expressed on effector T cells, was still co-expressed on Db/CN54gag tetramer^+^ CD8^+^ T cells infiltrating genital tissue on day 28 post MA immunization, while downregulation of this marker was apparent on circulating Db/CN54gag tetramer^+^ CD8^+^ T cells (Fig. 4D). Thus MA immunization through the skin led to the instruction and accumulation of a memory pool of antigen-specific CD8^+^ T cells in the GT delineated by peripheral tissue homing/retention markers (CD49a^+^, CD69^+^, CD103^+^, CXCR3^+^) consistent with a tissue-resident memory T cell phenotype.

### 3.4 CD8^+^ T cell memory and recall responses in the genital tract after MA vaccination

**Fig. 5:**
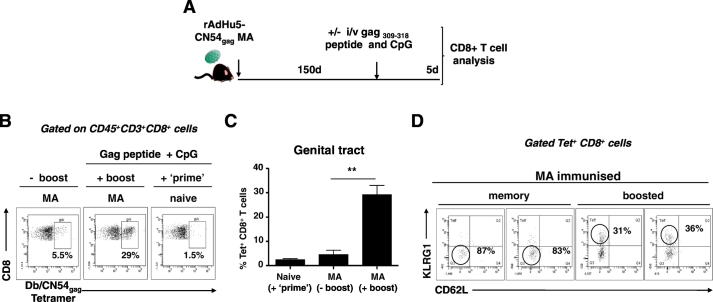
Durable CD8^+^ T cell memory and recall responses in the genital tract. (A) Schematic representation of the experimental protocol. 150 days after immunization with rAdHu5-CN54 gag by MA, groups of mice were either challenged intravaginally with antigen (20 μg HIV-1 CN54 gag_308–318_ peptide and 30 μg of CpG) or with PBS. A control group of naïve mice received the same antigen challenge dose intravaginally. Five days post-antigen challenge, isolated genital tract tissue was assessed for the frequency of effector and memory markers expressed on Db/CN54gag tetramer^+^ cells. (B) Dot plots of Db/CN54gag tetramer^+^ cells (derived from a CD45^+^ CD3^+^ CD8^+^ gate) from genital tract tissue isolated from (left panel; -boost) MA only immunized mice, (middle panel; + boost) MA immunized and intravaginal antigen challenged mice and (right panel) naïve mice challenged with antigen intravaginally. (C) Bar graph indicates the frequency of Db/CN54gag tetramer^+^ CD8^+^ cells (mean ± SEM) of each group of mice. (D) Frequency of effector/memory markers (KLRG1 and CD62L) expressed by Db/CN54gag tetramer^+^ CD8^+^ T cells isolated from the genital tract of memory mice (left), or mice boosted with antigen (HIV-1 CN54 gag_308–318_ peptide and CpG (right). Data are representative of two independent experiments (*n* = 4/group).

A fundamental requirement of vaccination is the generation of immunological memory and the ability to elicit CD8^+^ T cells that respond rapidly to antigen on secondary encounter. To that end, we investigated the recall capacity of Db/CN54gag tetramer^+^ CD8^+^ T cells present 150 days after a single vaccination with a candidate rAd5Hu HIV-1 CN54 gag vaccine delivered by MA (Fig. 5A). Over 5% of total CD8^+^ T cells isolated from the female GT of MA vaccinated mice stained for the Db/CN54gag tetramer at 150 days post vaccination (Fig. 5B). To determine the recall capacity of MA primed CD8^+^ T cells, mice were boosted (antigen challenged) intra-vaginally at day 150 post-vaccination with a CpG adjuvanted peptide corresponding to the immunodominant HIV-1 CN54 gag_308–318_ epitope (Fig. 5A). A significant increase in the frequency of Db/CN54gag tetramer^+^ CD8^+^ T cells was detected in GT tissue on day 5 following intra-vaginal antigen boost of vaccinated mice when cross compared with vaccinated but non-boosted mice (*p* < 0.05; Fig.5C). To determine the phenotype of responding Db/CN54gag tetramer^+^ CD8^+^ T cells, we selected CD62L and KLRG-1 to define; tissue resident effector memory cells as CD62L^−^ KLRG-1^−^ and effector T cells as CD62L^−^ KLRG-1^+^[23]. A substantial proportion of Db/CN54gag tetramer^+^ CD8^+^ T cells from the GT of antigen challenged female mice co-expressed high levels of KLRG-1 (> 30%) indicative of their effector phenotype (Fig. 5D) while, inversely, Db/CN54gag tetramer^+^ CD8^+^ T cells in the GT of MA immunized, but non-challenged mice, mostly retained an effector memory phenotype, lacking KLRG-1 or CD62L expression (Fig. 5D). Therefore, rAd5Hu-CN54gag MA vaccination generates antigen-specific CD8^+^ T cells that are retained over an extended time frame at the mucosal tissues and are able to rapidly expand in significant number upon locally administered antigenic challenge (Fig. 5D).

### 3.5 rAd5Hu-CN54gag MA immunization generates long-lived tissue resident memory CD8^+^ T cells within female genital tract tissue

**Fig. 6:**
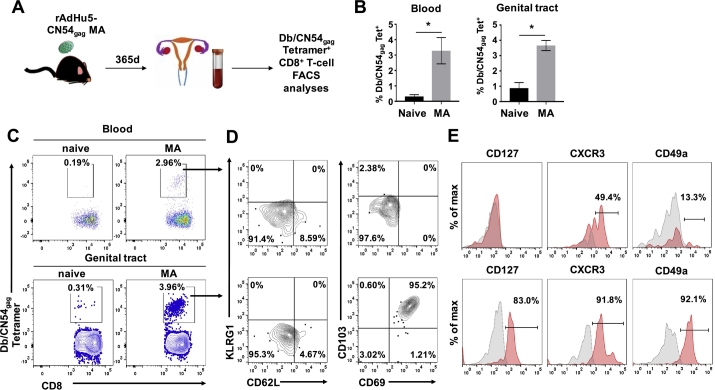
rAdHu5-CN54gag MA immunization generates long-lived, antigen-specific, tissue resident memory CD8^+^ T cells within female genital tract tissues. (A) Schematic of the experimental design to assess Db/CN54gag tetramer^+^ CD8^+^ T-cells in blood and genital tract of female mice 365 days after MA immunization with rAdHu5-CN54gag. (B) Bar graph indicates the frequency of Db/CN54gag tetramer^+^ CD8^+^ cells (mean ± SEM) enumerated in the blood and isolated genital tract tissue of naïve and rAdHu5-CN54gag MA immunized mice after 365 days. (C) Representative dot plots of Db/CN54gag tetramer^+^ CD8^+^ T cells assessed from blood (upper panels) and isolated genital tract tissue (lower panels) from mice enumerated in (B). (D) Representative contour plots showing the expression of KLRG1 and CD62L or CD103 and CD69 among Db/CN54gag tetramer^+^ CD8^+^ T cells isolated from blood (upper panels) or genital tract tissue (lower panels) of mice immunized with rAdHu5-CN54gag MA 365 days prior to analysis. (E) Representative histograms indicating the frequency of CD127, CXCR3 or CD49a expressing Db/CN54gag tetramer^+^ CD8^+^ T cells (red histograms) isolated from blood (upper panels) or genital tract (lower panels) of mice immunized with rAdHu5-CN54gag MA 365 days prior to analysis. Grey histograms represent staining with isotype control. Data are from one experiment (*n* = 4/group).

To assess the persistence of immunological memory upon MA immunization, we investigated the capacity of the live virus vectored MA platform to program long lived, antigen-specific memory cells in mice. For at least one year following rAd5Hu-CN54gag MA immunization, tractable HIV-1 CN54 gag_308–318_ CD8^+^ T cells were detected not only in the systemic compartment but also in the GT of immunized female mice (Fig. 6A and B). The Db/CN54gag tetramer^+^ CD8^+^ T cells retained their effector memory phenotype, as characterised by CD62L^−^ and KLRG-1^−^ expression of cells isolated from both the systemic and GT compartment. In contrast, high-level co-expression of markers of tissue residency, CD103, CD69, CD49a and CXCR3 was restricted to GT derived Db/CN54gag tetramer^+^ CD8^+^ T cells (Fig. 6C, D and E). Likewise, the majority of GT resident Db/CN54gag tetramer^+^ CD8^+^ T cells elicited by MA vaccination expressed CD127, a marker for long-term survival, which was absent from their circulating counterparts (Fig. 6E). Thus the bulk of antigen specific CD8^+^ T cells retained for up to one year in the GT tissue after MA vaccination are T_RM,_ programed with a receptor dependence on CXCR3 ligands for their homing/retention in GT tissue and IL-7 for tonic survival through CD127 (IL-7 receptor).

### 3.6 rAd5Hu-CN54gag MA immunization generates long-lived tissue resident memory CD8^+^ T cells within respiratory tract

**Fig. 7:**
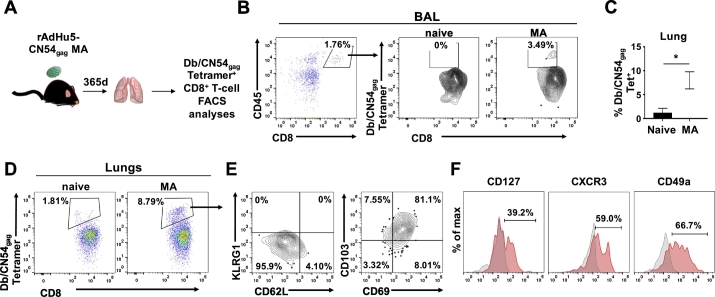
rAdHu5-CN54gag MA immunization generates long-lived, antigen-specific, tissue resident memory CD8^+^ T cells within the respiratory tract. (A) Schematic of the experimental design to assess Db/CN54gag tetramer^+^ CD8^+^ T-cells in BAL and lung parenchyma 365 days after MA immunization with rAdHu5-CN54gag. (B) Flow cytometric analysis showing Db/CN54gag tetramer^+^ cells among CD45^+^ CD8^+^ T cells isolated from BAL of naïve or MA immunized mice. (C) Bar graph indicates the frequency of Db/CN54gag tetramer^+^ CD8^+^ cells (mean ± SEM) enumerated in lung tissue of naïve and rAdHu5-CN54 gag MA immunized mice after 365 days. (D) Lung tissues isolated from naïve mice or rAdHu5-CN54gag MA immunized mice were assessed after 365 days for infiltration of Db/CN54gag tetramer^+^ CD8^+^ T cells by flow cytometry. (E) Representative contour plots showing the expression of KLRG1 and CD62L (left) or CD103 and CD69 (right) among Db/CN54gag tetramer^+^ CD8^+^ T cells isolated from lung tissues of mice immunized with rAdHu5-CN54gag MA 365 days prior to analyses. (F) Representative histograms indicating the frequency of CD127, CXCR3 or CD49a expressing Db/CN54gag tetramer^+^ CD8^+^ T cells (red histograms) isolated from lungs of mice immunized with rAdHu5-CN54gag MA 365 days prior to analysis. Grey histograms represent staining with isotype controls. Data are from one experiment (*n* = 4/group).

To extend our observations to another peripheral tissue, we investigated whether MA skin immunization led to the accumulation of long-lived Ag-specific CD8^+^ T cells in the respiratory tract (Fig. 7A). At one-year post immunization, both, BAL and lung tissue collected from immunized mice revealed a substantial population of Db/CN54gag tetramer^+^ CD8^+^ T cells (Fig. 7B, C and D). Such tetramer tracked CD8^+^ T cells recovered from the lung were simultaneously negative for KLRG-1 and CD62L (Fig. 7E) consistent with a memory phenotype and revealed a tissue resident phenotype, defined by co-expression of CD103 and CD69 that was imprinted by vaccination and consistent with their confinement to the lung parenchyma (Fig. 7E). Strikingly, Db/CN54gag tetramer^+^ CD8^+^ T cells from the lung demonstrated variable expression of CXCR3, CD49a and CD127 (Fig. 7F) and emphasised MA vaccination induced complementary CD69^+^ CD103^+^ CXCR3^hi^ and CD69^+^ CD103^+^ CXCR3^lo^ populations detected in the lung. Most probably the respective CD8^+^ T cell subsets reflect their programmed differentiation and localization in separate anatomic sites in the lung [24]. Collectively, these results indicate that skin immunization with an Ad viral vectored vaccine generated antigen-specific CD8^+^ T cell immunity engendering long-lasting memory responses not only in the systemic compartment, but more importantly, in the peripheral mucosal tissues, where heterogeneous CD8^+^ T cell subsets in a layered response could play a crucial role to control pathogenic infection.

### 3.7 Vaccine elicited CD8^+^ T cells retain functionality one-year post MA vaccination

**Fig. 8:**
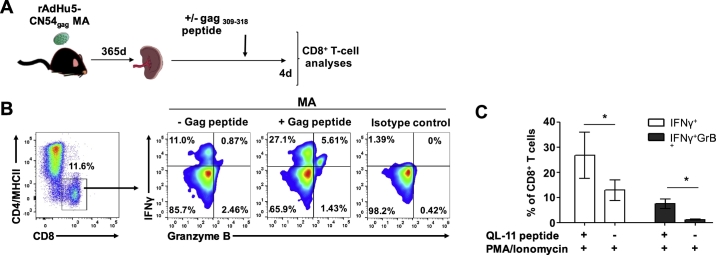
Antigen–specific CD8^+^ T cells retain functionality one-year post MA vaccination. (A) Schematic of the experimental design; single cells from the spleen of MA immunized mice 365 days post-vaccination were sensitized in vitro with an HIV-1 CN54 gag_308–318_ peptide for 4 days or left untreated prior to intracellular cytokine staining performed for IFNγ and Granzyme B in the presence of PMA and Ionomycin. (B) gated CD8^+^, MHCII^−^, CD4^−^ cells (left) were further assessed for poly-functionality by flow cytometry. Biaxial plots demonstrate simultaneous detection of IFNγ and Granzyme B (right). Isotype control staining is also shown (far right). (C) Bar graph indicates the frequency of Db/CN54gag tetramer^+^ CD8^+^ cells (mean ± SEM) enumerated from data shown in (B). Data are from one experiment (*n* = 4/group).

Although the generation and maintenance of immunological memory is a prerequisite of a vaccine, functional competence is essential during a recall response to immunological challenge. To that end, one year post MA vaccination, splenic CD8^+^ T were sensitized in vitro with cognate HIV-1 CN54 gag_308–318_ peptide for 4 days to expand cells (Fig. 8A) and the ability of responders to produce IFNγ, an antiviral cytokine and also mediators of cytotoxic effector function (Granzyme B) was assayed by intracellular cytokine staining and flow cytometry after stimulation with PMA and Ionomycin (Fig. 8B). Background levels of IFNγ and Granzyme B were determined in the absence of peptide stimulation (peptide control). Antigen specific CD8^+^ T cells sensitized with HIV-1 CN54 gag_308–318_ peptide derived from MA vaccinated mice generated high frequency monofunctional IFNγ^+^ Granzyme B^−^ cells compared to un-stimulated controls (*p* < 0.05). Similarly, high frequency polyfunctional, IFNγ^+^ Granzyme B^+^ cells were detected following in vitro expansion when compared with peptide-unstimulated controls (p < 0.05) (Fig. 8B and C). These data establish that MA vaccinated mice produced long-lived antigen specific cells that retain T-cell effector functionality.

## 4 Discussion

The success of live viral vectored vaccines in eliciting potent cellular immune responses lies in their capacity to efficiently infect (or transduce) antigen presenting cells, directly [25] or indirectly [26], [27] and prime antigen-specific CD8^+^ T cells without the need of an adjuvant [28], [29]. The challenge has been to retain the immunogenicity of these live vaccines when lyophilized or dried in a MA platform (to eliminate cold chain storage) and at the same time induce a multi-layered memory CD8^+^ T cell response providing immune surveillance at each immune barrier; mucosal, central lymphoid and vascular against invading pathogens. Here we report the first evidence that fabrication of a dried MA formulated from a dissolvable polymer containing a live recombinant AdHu5 vector when delivered to skin has the capacity to elicit long-lived antigen–specific CD8^+^ T cells at the mucosal barrier. Specifically, we reveal that CD8^+^ T_RM_ cells programmed with antiviral effector function were induced and retained both in the genital and respiratory tracts for at least one year following vaccination with an exemplar (rAdHu5 HIV-1 gag) vaccine released from the matrix of a rapidly dissolvable MA.

Prior studies have successfully demonstrated the ability of dried MA virus vectored vaccines to instruct memory CD8^+^ T cell responses in secondary lymphoid tissues [14], [15], [16], [30] and also programme CD8^+^ T_EM_ cells that patrol non-lymphoid tissues [16]. Indeed it is widely accepted that T cells activated at any site, whether through local or systemic priming, can gain the capacity to circulate through almost all tissues in the body [31], [32] and be recruited to sites of virus infection. However, for viruses that enter via the epithelial surfaces, such as HIV, herpes simplex, influenza virus and Zika virus, the deployment of T_RM_ cells that permanently reside within the epithelia following priming may be critical for rapid initial virus control, before other memory T cell populations are recruited to prevent systemic virus spread [33], [34]. In that regard, our data extend the known capabilities of the MA delivery platform by demonstrating that MA vaccination through the skin promotes priming and retention of T_RM_ cells in the female genital tract and in the lung.

Although it has been reported that local antigen is necessary to drive the generation of T_RM_ cells in mucosal tissues [33], [35], [36] previous studies nonetheless reported that systemic immunization with non-replicating viral vectors induced CD8^+^ T cell responses in the female genital tract [37], [38], [39]. However, unlike those reports where vaccine delivery required traditional needle-based immunization; our data provide evidence that MA immunization engenders retention of antigen-specific CD8^+^ T_RM_ cells without the necessity for vaccine reconstitution, hypodermic needle delivery or cold chain vaccine vector storage. Of caution, prior studies [37], [38], [39] left open the possibility that memory CD8^+^ T cells reported as isolated from the genital tract following systemic immunization may have been derived from the vasculature during tissue disaggregation. To exclude that possibility, we comprehensively phenotyped antigen-specific CD8^+^ T cells tracked by tetramer in the genital tract for peripheral tissue retention markers that are signatures of CD8^+^ T_RM_ cells. At 150 and 365 days after MA immunization, tetramer-specific CD8^+^ T cells in the genital tract uniformly expressed surface CD103, an α_E_ integrin binding E-cadherin, highly expressed on epithelia [40] and also expressed CD69, an antagonist to sphingosine 1-phosphate receptor 1 (S1PR1) that regulates egress from tissues [41]. These data strongly suggest rAdHu5 vectored MA immunization promotes the differentiation and retention of a subpopulation of primed CD8^+^ T cells in female genital mucosa. Consistent with their barrier tissue localisation, a high frequency of CD8^+^ T_RM_ cells induced by MA immunization in the genital tract and lungs co-expressed CD49a, a biomarker that defines T_RM_ cells poised with cytotoxic potential [42], [43]. The fact that tetramer^+^ CD103^+^ CD69^+^ CD8^+^ T cells were retained for up to one year in mucosal tissues in the absence of any booster vaccination may suggest that once this memory population is programmed by MA vaccination, that local signals promote their long term survival.

Cutaneous MA immunization also elicited high frequency antigen-specific CD103^+^ CD69^+^ CD8^+^ T_RM_ cells that were localized in the lung. Most unexpectedly, two distinct populations of CD103^+^ CD69^+^ CD8^+^ T_RM_ cells, identified as CXCR3^LO^ and CXCR3^HI^were retained in the lung one-year post MA immunization, which was not apparent among the CD8^+^ T_RM_ cells retained in genital tract tissue that were uniformly CXCR3^HI^CD49a^Hi^. The functional consequences of distinct subsets of lung resident memory CD8^+^ T cells induced by MA immunization remains to be clarified. In this regard, MA induced CXCR3^LO^ CD8^+^ T_RM_ cells might reside in a distinct niche within the lung interstitium as reported after intranasal immunization [35] and a CXCR3^HI^ CD8^+^ T_RM_ subset within the lung airway [35]. This remains to be clarified, though for now however, the data offer strong support for the idea that MA immunization elicits overlapping CD8^+^ T_RM_ populations that patrol the lungs possibly for rapid and delayed mobilization [35].

Understanding the long-term capacity of MA elicited memory CD8^+^ T cells to respond to recall antigen is pivotal to enable further development and future clinical deployment of this vaccination platform. To that end, memory CD8^+^ T cells generated one year post MA vaccination maintained Granzyme B expression mediating their ex vivo cytotoxicity, in a similar manner to antigen-specific effector CD8 + T cells [44], [45]. Rapid delivery of cytotoxic molecules represents only one potential mechanism to eliminate anamnestic infections. Here, we also show that a large proportion of MA elicited HIV-1 gag_308–319_ specific memory CD8^+^ T cells also released IFNγ as an antiviral effector, or co-expressed both IFN-γ and Granzyme B in a response to peptide re-stimulation, confirming their desirable T-cell effector poly-functionality. Our study also demonstrated a durable ability of MA induced CD8^+^ T cells to respond to secondary antigen challenge in the vagina with an apparent 5 to 6-fold increase in the frequency of Db/tetramer^+^ CD8^+^ T cells in the genital tract tissues. This expansion, likely involved recruitment of KLRG-1^+^ short term effectors from the draining lymph nodes to the genital tract with enhanced kinetics following antigen challenge at that site, as at a minimum, one third of Db/tetramer^+^ CD8^+^ T cells in the genital tract of antigen challenged mice displayed an effector phenotype (KLRG-1^+^ CD62L^−^). In that regard, enrichment of effector CD8^+^ T cells following challenge is consistent with earlier reports of T cell mobilization from the iliac lymph nodes to the vagina after vaginal antigen/virus challenge [38], [46].

Our study lends strong support for the premise that MA vaccination of an accessible tissue, the skin, enables rapid vaccine dissolution and elicits a long-lived KLRG-1^−^ CD62L^−^ CD103^+^ CD69^+^ CXCR3^+^ T_RM_ memory population expressing CD49a, programmed for rapid antiviral deployment in the genital and respiratory tract that is complemented by lymphoid tissue based CD8^+^ T_CM_ and patrolling CD8^+^ T_EM_ cells that in aggregate can provide a multi-layered immune response to pathogen challenge.

## 5 Conclusions

Overall, this study shows that the dissolving MA delivery platform offers an attractive approach to administer rAdHu5 virus vectored vaccines to generate long-lived antigen specific CD8^+^ T cells that reside within the barrier surfaces, retaining their T-effector poly-functionality and demonstrating durable ability to respond to secondary antigen challenge. This property of the MA-mediated vaccine delivery platform, to programme the expression of peripheral tissue homing and retention molecules on the responding HIV-specific CD8^+^ T cells, has a potential to substantially increase the protective efficacy at the entry point of this and other pathogens. This attribute, combined with the significant logistical advantages offered by microneedle technology (cold chain independence, increased safety due to elimination of hazardous sharps, ease of administration and likely increased patient compliance), bode well for advancing deployment of the MA vaccine technology in the clinic, not only restricted to the context of infectious diseases, but also in other therapeutic applications. Going forward, transition to the clinic will require a GMP manufacturing process and GMP packaging to protect the MAs in storage and distribution. Additionally, an applicator may prove beneficial for MA delivery by trained healthcare workers in the first instance. Nonetheless, incorporation of a pressure sensing film may enable self-application in the future by indicating the appropriate pressure to apply for effective MA delivery.

## Author contribution

M.Z, P.D.B, C.H and L.S.K designed the research; M.Z, P.D.B., C.H, P.K, B.I.Y, C.C and L.A.O.N performed the research; S.-Y.K contributed reagents/materials; M.Z, P.D.B., C.H, P.K, B.I.Y, C.C, L.A.O.N and L.S.K analyzed the data and M.Z and L.S.K wrote the paper.

## Conflict of interest statement

S.-Y.K is a shareholder in TheraJect that holds the patent rights for dissolvable microneedle arrays for vaccine delivery.

## Funding

This work was funded by the Bill and Melinda Gates Foundation, Seattle, WA, grant number 38639 and a European Union Marie Curie Initial Training Network (Univacflu) award, grant number 607690.
